# Development and Clinical Validation of a Digital Eye-Tracking–Based Cover Test for Ocular Misalignment

**DOI:** 10.1016/j.xops.2026.101261

**Published:** 2026-05-28

**Authors:** Eduardo Esteban-Ibañez, David Solanas, Marta Lacort-Beltrán, Marina Vilella, Xian Pan, Olimpia Castillo, Marta Ortín, Victoria Pueyo

**Affiliations:** 1 Aragon Institute for Health Research (IIS Aragón), Zaragoza, Spain; 2 Ophthalmology Department, Miguel Servet University Hospital, Zaragoza, Spain; 3 Universidad de Zaragoza, I3A, Zaragoza, Spain; 4 Department of Microbiology, Pediatrics, Radiology, and Public Health, Faculty of Medicine, Universidad de Zaragoza, Zaragoza, Spain

**Keywords:** Strabismus, Cover test, Sensorimotor examination, Eye tracking, Digital device

## Abstract

**Purpose:**

To develop and clinically validate the DIVE Cover Test (DCT), a portable eye-tracking–based digital implementation of the cover test, and to assess its diagnostic accuracy, agreement with the clinical reference standard (classic cover test [CCT]), and repeatability across cover–uncover test (CUT) and alternate cover test (ACT) phases and across horizontal and vertical axes.

**Design:**

A prospective clinical validation study.

**Participants:**

Sixty-eight participants aged 3 to 74 years, including healthy controls and patients referred for strabismus evaluation.

**Methods:**

Participants underwent 2 repetitions of the DCT and 2 independent CCT examinations performed by experienced clinicians at near distance (65 cm). The order of the 4 assessment blocks was randomized. Diagnostic performance (sensitivity/specificity) for detecting ocular misalignment was calculated using CCT classification as reference. Agreement and repeatability were evaluated using Bland–Altman mean differences and 95% limits of agreement (LoA), intraclass correlation coefficients (ICCs[2,k]), and Pearson correlations, separately for CUT versus ACT and horizontal versus vertical components.

**Main Outcome Measures:**

Sensitivity and specificity for detection of ocular misalignment; intermethod agreement between DCT and CCT; test–retest repeatability of the DCT; and interobserver agreement of the CCT.

**Results:**

For detecting any deviations, the DCT achieved 85% sensitivity and 98% specificity in CUT, and 92% sensitivity and 87% specificity in ACT. Agreement between DCT and CCT was strongest for horizontal deviations, particularly during ACT (mean difference –1.3 prism diopters [PD]; LoA –8.0 and 5.4 PD). Intermethod ICCs were high for horizontal deviations (CUT 0.93; ACT 0.96) and moderate-to-high for vertical deviations (both phases 0.81). The DCT test–retest repeatability was excellent (ICC ≥0.93 across axes and phases), with narrow LoA (±3.8 to ±4.6 PD). Classic cover test interobserver agreement was also high (ICC ≥0.95 across phases and axes).

**Conclusions:**

The DCT demonstrates high diagnostic accuracy, strong agreement with the clinical reference standard, and excellent repeatability. Phase-specific (CUT vs. ACT) and axis-specific (horizontal vs. vertical) analyses support the DCT as a practical, portable, and objective tool for quantifying ocular misalignment in clinical settings.

**Financial Disclosure(s):**

Proprietary or commercial disclosure may be found in the Footnotes and Disclosures at the end of this article.

Ocular misalignment, which affects 3% to 6% of the general population,^1^, ^2^, ^3^, ^4^ is a clinically significant condition that can lead to amblyopia, diplopia, stereopsis reduction, and impaired binocular vision if not detected and treated early.^4^^,^^5^ Accurate and timely quantification of ocular deviations is essential for determining appropriate therapeutic interventions—ranging from optical corrections and occlusion or visual therapy to surgical procedures—and for monitoring patients over time, often across multiple follow-up sessions over several years.^6^^,^^7^

The prism-cover test, considered the reference standard for evaluating ocular alignment,^8^ consists of 2 phases: cover–uncover and alternate cover. However, this method is inherently subjective, relying heavily on the examiner’s expertise and patient cooperation.^6^^,^^9^^,^^10^ This dependence may introduce variability and make the test particularly challenging in pediatric populations. The use of prism bars close to the eye to measure deviation can be intimidating for children, and the repetitive, unengaging nature of the test may reduce attention and compliance. Additionally, the lack of objective digital recordings hinders the ability to perform consistent, longitudinal comparisons of a patient's deviation over time.

In recent years, advances in digital technologies have led to the development of new tools for the assessment of ocular deviation,^11^ primarily based on video-oculography^7^^,^^12^, ^13^, ^14^, ^15^ and eye-tracking approaches implemented on computer- or screen-based systems,^16^, ^17^, ^18^, ^19^ or integrated into virtual reality headsets.^20^, ^21^, ^22^, ^23^ While these approaches have shown promising results in research settings, many existing systems are laboratory-based, require complex setups, or lack sufficient portability, which limits their practical applicability in routine clinical environments. As a result, there remains a gap between technological advances in ocular alignment assessment and their translation into practical, scalable tools suitable for everyday clinical use.

Eye-tracking technology nonetheless offers a promising solution to many of the limitations of traditional strabismus assessment.^16^, ^17^, ^18^, ^19^ Portable eye-tracking systems, such as DIVE, have been specifically designed for clinical use, allowing objective ocular alignment assessment outside laboratory settings. These devices are noninvasive, engaging, and have already been validated for assessing visual function—including oculomotor control, visual acuity, and contrast sensitivity—in infants as young as 6 months.^24^, ^25^, ^26^ These systems enable automated detection of eye movements during cover test sequences, minimizing the need for specialist intervention and transforming a clinical procedure into a more game-like experience. This not only improves the patient’s engagement, especially in younger children, but also offers the potential for more predictable, objective, and quantifiable measurements. However, for such digital tools to be clinically meaningful, they must demonstrate adequate repeatability and agreement with established clinical methods across different test conditions and measurement axes.

The purpose of this study was to develop and validate a novel cover test based on eye-tracking technology—the DIVE Cover Test (DCT)—and to compare its repeatability with the classic cover test (CCT) conducted by 2 experienced clinicians. Specifically, we evaluated the repeatability and agreement of both methods during cover–uncover and alternate cover testing, assessing horizontal and vertical components separately. We hypothesized that this digital method would provide reliable and objective measurements of ocular deviation, with agreement and repeatability comparable to those of the traditional clinical assessment.

## Methods

### Participants

This study was conducted at the Pediatric Ophthalmology Unit of the Hospital Universitario Miguel Servet, Zaragoza, Spain. We included both healthy controls and patients referred for strabismus evaluation. Control subjects were recruited from patients attending the unit for routine ophthalmic examination and were confirmed to have normal ocular alignment and no ocular or neurological conditions affecting binocular vision.

Inclusion criteria were age over 3 years and the ability to maintain fixation on a Lang Cube target. Lack of cooperation during the cover test was not considered an exclusion criterion, as the DCT is designed to be usable even in low-cooperation settings. Participants with manifest deviations exceeding 25 prism diopters (PD) were excluded to minimize the impact of extreme values on agreement analyses, as large deviations may magnify apparent discrepancies between methods due to the discrete nature of prism-based measurements at higher ranges. No exclusion was applied based on refractive error; however, participants with ocular pathologies other than strabismus that could interfere with ocular alignment measurements were excluded. For the control group, the absence of ocular deviation or conditions affecting binocular vision was additionally required. Participant sex was recorded but was not used as a stratification variable in the analyses.

All procedures adhered to the tenets of the Declaration of Helsinki. The study was approved by the Institutional Review Board, Comité Ético de Investigaciones Clínicas de Aragón (reference: EC22/00002). Written informed consent was obtained from all adult participants. For children, written informed consent was obtained from a parent or legal guardian. Participants aged 12 to 18 years also provided written assent in addition to parental consent.

### Exploration Procedures

**Figure 1:**
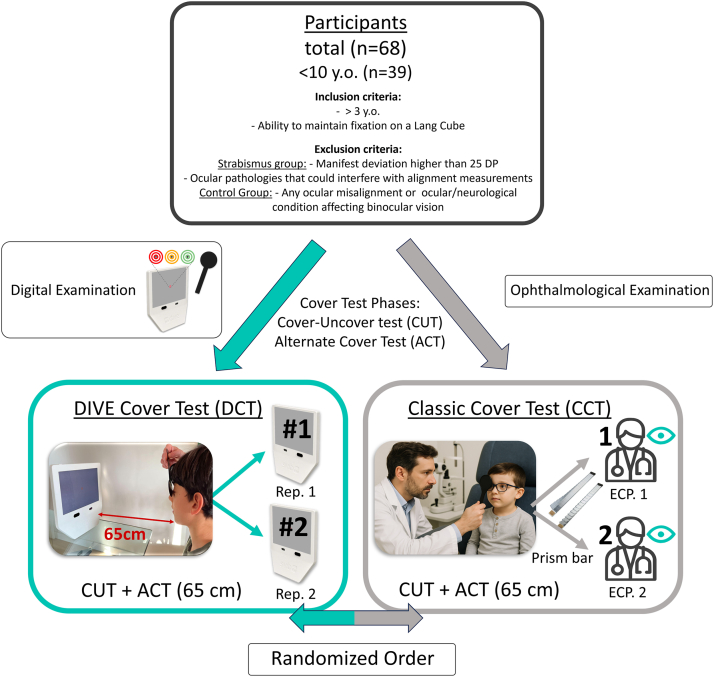
Study workflow and examination protocol. Schematic overview of the experimental protocol. Each participant underwent a digital and clinical ophthalmic examination, including 2 repetitions of the DIVE Cover Test (DCT) and 2 independent assessments with the classic cover test (CCT) performed by 2 experienced eye care practitioners (ECPs). Both tests were conducted at a distance of 65 cm and included 2 phases: CUT and ACT. The order of the block of assessments was randomized for each participant to minimize potential order effects and fatigue. ACT = alternate cover test; CUT = cover–uncover test.

All participants underwent a standardized evaluation protocol consisting of 2 types of cover tests: the automated DCT and the CCT, as part of a complete ophthalmic examination. Each subject completed 2 repetitions of the DCT and was independently examined by 2 experienced clinicians performing the CCT. The order of assessment blocks was randomized for each participant to minimize potential order effects, reduce fatigue, and eliminate examiner-related bias. This examination sequence is illustrated in Figure 1.

#### Ophthalmic Examination

All participants underwent a complete ophthalmic assessment, including visual acuity assessment under habitual refractive correction (using age-appropriate optotypes), followed by ocular alignment and motility testing performed prior to cycloplegia using both the CCT and the DCT, and subsequently cycloplegic refraction (to determine best-corrected visual acuity) and fundus examination. Binocular function (including fusion) was assessed in control participants to confirm normal binocular vision, although it was not systematically recorded in patients with strabismus.

Ocular alignment was evaluated in primary gaze at near distance (65 cm), under habitual refractive correction, using 2 CCT phases: the cover–uncover test (CUT) and the alternate cover test (ACT). Two experienced eye care practitioners (V.P. and E.E.-I.) independently performed both CUT and ACT using prism bars for quantification. Results were recorded independently for each observer using standardized scoring sheets.

#### DIVE Device Examination

All patients also completed testing with the DCT, implemented on a DIVE, a digital device equipped with eye-tracking technology. The system includes a 12-inch high-resolution display (2160 × 1440 pixels) corresponding to a visual angle of 28.46° horizontally and 19.19° vertically at a viewing distance of 65 cm (at which the tests are carried out). Eye tracking was performed using a Tobii Eye Tracker 5L, sampling at 120 Hz. According to manufacturer specifications, binocular accuracy and precision were 0.6° and 0.25°, respectively, while monocular values were 0.8° and 0.34°.

Screen brightness was calibrated weekly using a Datacolor SpyderX device (Datacolor Co.) with gamma setting of 2.2,^27^ a white point of 6500 K, and a target luminance of 120 cd/m^2^.

A nine-point binocular calibration was performed before each test. During this step, participants were asked to fixate on a cartoon stimulus accompanied by a sound cue that appeared sequentially at 9 predefined locations across the display. After calibration, a validation procedure using the same nine-point layout was performed to verify tracking accuracy. Participants again fixated on each stimulus while the system evaluated the quality of gaze mapping at every position.

The calibration–validation sequence was repeated, when necessary, until at least 6 valid calibration points were obtained, and the overall calibration quality reached a minimum score of 3 out of 5, according to the device’s internal quality metric.

##### DIVE Cover Test (DCT)

**Figure 2:**
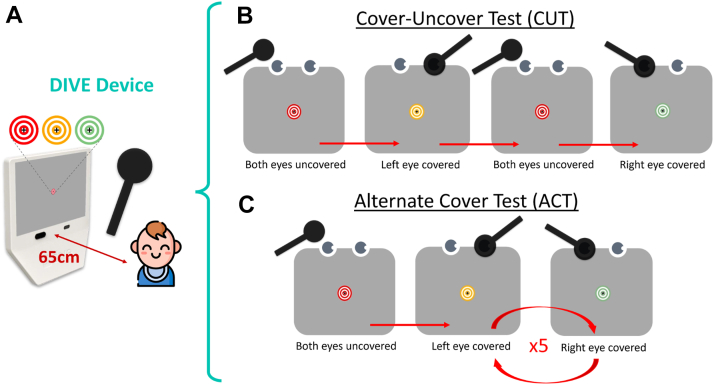
Implementation of the digital DIVE Cover Test (DCT). (**A**) The DIVE device uses an eye-tracking system to monitor gaze while participants view stimuli at a fixed distance of 65 cm. (**B**) The cover–uncover test (CUT) includes a single occlusion per eye, alternating with binocular viewing. (**C**) The alternate cover test (ACT) consists of 5 consecutive occlusions with alternating monocular coverage, without binocular recovery between occlusions. Stimulus color cues indicate the occlusion side to the examiner, and continuous gaze tracking is maintained throughout the sequence.

The DCT is a digital, eye-tracking–based implementation of the standard cover test protocol (represented in Fig 2). It reproduces both the CUT and the ACT, designed to be performed in primary gaze at a near-viewing distance of 65 cm, under habitual refractive correction (Fig 2A). During the test, participants were instructed to maintain fixation on a central circular stimulus, shaped as a target with a central cross, displayed on the screen while monocular occlusions were manually performed by the examiner according to the visual and verbal instructions provided by the device.

To allow continuous binocular gaze tracking during occlusion periods, a visible-light filter occluder was used. This occluder blocks visible light, enabling proper occlusion from the participant’s point of view, while remaining transparent to infrared light, thus permitting uninterrupted signal acquisition by the eye tracker. The stimulus displayed on the screen changed color to indicate which eye should be occluded—right, left, or neither—serving as a cue for the examiner to manually perform the occlusion in synchrony with the programmed test sequence. This visual cue was accompanied by an auditory signal and spoken instruction to facilitate correct timing of the occlusion. Each CUT sequence consisted of a single occlusion of each eye, followed by a return to binocular viewing (Fig 2B). In contrast, the ACT phase involved 5 alternating occlusions between the 2 eyes, with no binocular viewing periods between them (Fig 2C).

Throughout the procedure, binocular gaze position was continuously recorded along both the horizontal and vertical axes. Eye deviation was automatically calculated based on differences in eye position during occluded and nonoccluded phases of the test. Data from both eyes were analyzed, with ocular deviations quantified separately for the horizontal and vertical axes. Eye position differences were initially computed in degrees of visual angle and subsequently converted to PD using standard geometric conversion.

##### Results Report

**Figure 3:**
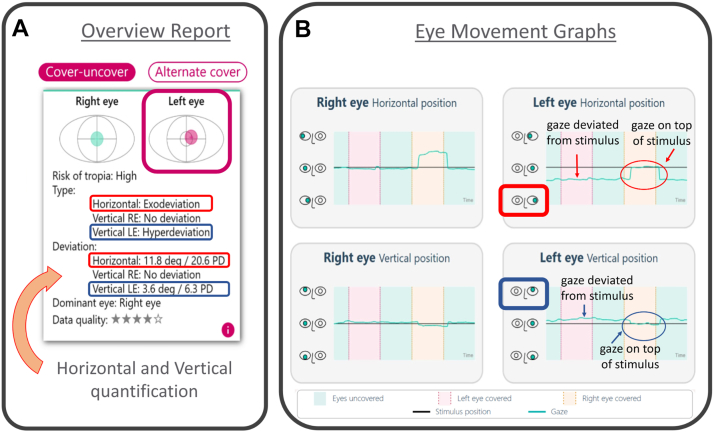
Output reports generated by the DIVE Cover Test. (**A**) Overview report displaying the classification of horizontal and vertical deviations for each eye and the corresponding quantitative measurements in degrees and prism diopters (PD). (**B**) Eye movement graphs show horizontal and vertical eye positions over the course of the test for each eye. Color-coded backgrounds indicate the occlusion phase: binocular viewing, left-eye occlusion, and right-eye occlusion. Deviations from fixation during occlusion and refixation movements upon uncovering are visible, enabling visual verification of alignment shifts.

Following the completion of each DCT session, the system automatically generated a detailed report, as displayed in Figure 3. This report included the quantitative measurement of ocular deviation in both the horizontal and vertical directions (shown in Fig 3A).

Additionally, a visual representation of the eye movement traces was provided, showing the horizontal and vertical gaze positions over time for both eyes (shown in Fig 3B). These graphs were annotated with color-coded bars indicating the occlusion status during each time segment: right-eye occlusion, left-eye occlusion, or binocular viewing. This visual feedback allowed both clinicians and researchers to assess the quality and dynamics of the eye movements during each phase of the test.

#### Validation Procedure: Comparison between DCT and CCT

To evaluate the clinical performance of the DCT, we designed a comparative protocol using the CCT as the reference standard. Each participant completed both tests—DCT and CCT—during the same clinical visit and under standardized conditions, including identical testing distance (65 cm) and room lighting.

The CCT was performed independently by 2 experienced eye care practitioners (V.P. and E.E.-I.), with a brief rest interval between evaluations. Each examiner conducted both the CUT and the ACT in accordance with routine clinical procedures. Ocular deviations were measured horizontally and vertically in prismatic diopters using prism bars, and each examiner recorded their results on individual datasheets to ensure examiner blinding and eliminate mutual influence. In addition, examiners were masked to the results of the DCT at the time of clinical assessment. This arrangement enabled the evaluation of interobserver variability.

The DCT was also performed twice per participant. To minimize learning effects, order effects, and fatigue-related artifacts, the order of the 2 assessment blocks—DCT (2 repetitions) and CCT by eye care practitioners (2 examiners)—was randomized across participants. Within the DCT block, the 2 repetitions were separated by a brief interval requiring participant repositioning prior to the second measurement. Because the DCT is fully automated and does not require subjective interpretation during the test itself, repeated administration enabled the assessment of intrasubject variability and test–retest reliability under equivalent conditions.

This balanced and randomized design provided the necessary framework to compare deviation measurements between digital and clinical methods, while controlling for order effects and observer influence.

### Data Analysis

All data were processed and analyzed using R 4.5.1 (R Foundation for Statistical Computing). Descriptive statistics were computed for horizontal and vertical ocular deviation measurements, separately for CUT and ACT conditions. Given the sample size, the continuous nature of the outcome variables, and the robustness of selected methods to moderate deviations from normality, parametric approaches were applied throughout the analyses.^28^

For diagnostic accuracy analyses, ocular alignment was classified as normal or abnormal using the CCT performed by the first examiner (V.P.) as the reference standard. A deviation was considered abnormal when the measured magnitude was ≥4 PD in either the horizontal or vertical axis within the corresponding test phase.

Agreement analyses included Bland–Altman plots^29^ to estimate mean differences and 95% limits of agreement (LoA) between DCT and CCT measurements, between repeated DCT trials, and between the 2 CCT observers. In addition, regression analyses of the differences between methods (DCT and CCT) against their mean values were performed to assess the presence of proportional bias in the Bland–Altman analysis, separately for each test phase and axis. Pearson correlation coefficients were also computed to assess linear associations, not as a substitute for agreement analyses, between repeated measurements within each method and between DCT and CCT.

Intraclass correlation coefficients (ICCs)^30^ were calculated to quantify agreement and repeatability. A two-way random-effects model with average measures (ICC[2,k]) was used throughout; absolute agreement was applied for intramethod analyses, while consistency was used for intermethod comparisons to account for potential systematic differences between techniques.

All analyses were conducted independently for CUT and ACT, and for horizontal and vertical deviations, to preserve clinical interpretability and accuracy.

## Results

A total of 68 participants, aged between 3 and 74 years (15 ± 14 years; 39 under 10 years of age), were enrolled in the study, comprising 37 females and 31 males. For the CUT, a total of 68 participants were assessed, of whom 42 were classified as having normal ocular alignment based on the CCT, and 26 were diagnosed with strabismus. In the ACT condition, 59 participants were examined, with 23 presenting normal alignment and 36 showing some form of ocular deviation, either manifest or latent. The lower number of ACT assessments likely reflects limited cooperation in younger children, owing to its longer duration and greater number of occlusions. In these cases, measurements were obtained but subsequently excluded from the analysis due to insufficient reliability, as determined by the device’s internal quality control criteria.

### Strabismus Detection and Diagnostic Performance of the DCT

The DCT identified both horizontal and vertical ocular deviations in the CUT and ACT phases. In the CUT phase, the CCT detected 26 abnormal cases, including 10 esotropias, 7 exotropias, and 9 mixed strabismus. In the ACT phase, the number of abnormal cases increased to 36, consisting of 12 esodeviations, 16 exodeviations, and 8 mixed deviations.

Using the CCT classification determined by examiner 1 (V.P.) as reference standard, the diagnostic accuracy of the DCT is summarized in Table 1, showing detailed sensitivity and specificity values for each test phase and deviation type, including 95% confidence interval (CI).

**Table 1:** Diagnostic Accuracy of the DIVE Cover Test (DCT) Using the Classic Cover Test (CCT) Performed by Examiner 1 as Reference Standard for Both Test Phases: CUT and ACT

Test Phase	Deviation Type	Deviated (n)	Nondeviated (n)	Sensitivity (%)	95% CI (%)	Specificity (%)	95% CI (%)
CUT	Any Strabismus	26	42	85%	[65%–96%]	98%	[87%–100%]
	Horizontal Strabismus	26	42	81%	[61%–93%]	98%	[87%–100%]
	Vertical Strabismus	9	59	89%	[52%–100%]	90%	[79%–96%]
ACT	Any Deviation	36	23	92%	[78%–98%]	87%	[66%–97%]
	Horizontal Deviations	36	23	89%	[74%–97%]	87%	[66%–97%]
	Vertical Deviations	8	51	88%	[47%–100%]	84%	[71%–93%]

ACT = alternate cover test; CI = confidence interval; CUT = cover–uncover test.

Sensitivity and specificity are presented with exact binomial 95% confidence intervals.

Overall, diagnostic performance was high in both phases. In the CUT condition, sensitivity was already high, but the most prominent feature was the very high specificity. Conversely, in the ACT phase, sensitivity reached its highest values while specificity remained within a similarly strong range.

### Ocular Deviation Magnitudes across Methods

Deviation magnitudes measured with the CCT and DCT were compared for both test phases.

In the CUT phase, the mean horizontal deviation recorded by the CCT was 13.7 PD [95% CI: 11.1–16.2] while the DCT yielded horizontal deviation with a mean of 13.3 PD [95% CI: 8.2–18.3]. Vertical deviations in the CUT condition averaged 2.6 PD [95% CI: 0.7–4.5] and 5.8 PD [95% CI: 3.2–8.3] for CCT and DCT, respectively.

During the ACT phase, horizontal deviations measured by the CCT had a mean of 9.4 PD [95% CI: 6.5–12.2] while the DCT registered 11.2 PD [95% CI: 7.2–15.2]. Vertical deviations were 1.4 PD [95% CI: 0.2–2.7] for the CCT and 4.4 PD [95% CI: 2.5–6.4] for the DCT.

These results indicate broadly comparable deviation magnitudes between methods across both test phases, with similar central tendency and dispersion.

### Agreement Analyses: DCT vs. CCT Interobserver and Test–Retest

#### Bland–Altman Analyses

**Figure 4:**
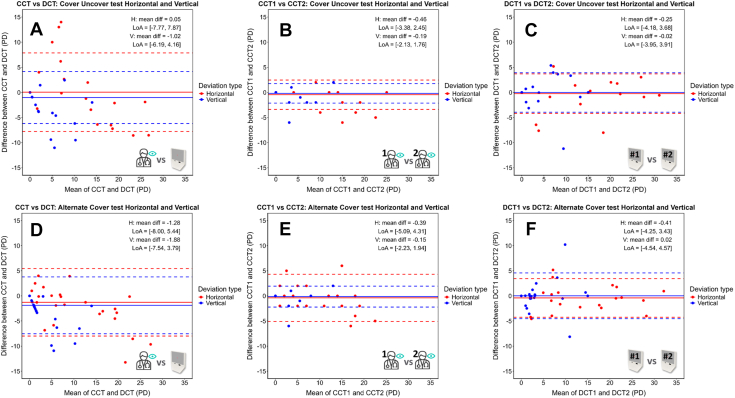
Bland–Altman plots showing agreement in deviation measurements for horizontal (red) and vertical (blue) axes. Top row: CUT phase; bottom row: ACT phase. Each row includes comparisons between (**A**, **D**) DCT and CCT, (**B**, **E**) the 2 CCT observers (ECP1 vs. ECP2), and (**C**, **F**) repeated DCT sessions (DIVE#1 vs. DIVE#2). Solid lines indicate mean differences; dashed lines represent the 95% limits of agreement, following the color scheme for each deviation type. CCT = classic cover test; CUT = cover–uncover test; DCT = DIVE Cover Test; ECP = eye care practitioner; LoA = limits of agreement; PD = prism diopters.

Bland–Altman analyses were conducted to evaluate agreement between the DCT and CCT, between the 2 CCT observers, and between repeated DCT sessions. These comparisons were performed separately for CUT and ACT, and for both horizontal and vertical axes. The resulting plots are shown in Figure 4.

In the CUT phase, the comparison between CCT and DCT (Fig 4A) resulted in a mean difference of 0.1 PD in the horizontal axis, with 95% LoA of –7.8 and 7.9 PD, and –1.0 PD in the vertical axis (LoA: –6.2 and 4.2 PD). Regression analysis of the differences against the mean values demonstrated a statistically significant proportional bias for both horizontal (slope = –0.21, *P* = 0.004) and vertical deviations (slope = –0.53, *P* < 0.001). The interobserver analysis for the CCT (Fig 4B) showed a horizontal mean difference of –0.5 PD (LoA: –3.4 and 2.5 PD) and a vertical difference of –0.2 PD (LoA: –2.1 and 1.8 PD). For the DCT (Fig 4C), test–retest agreement yielded a horizontal mean difference of –0.3 PD (LoA: –4.2 and 3.7 PD) and a vertical mean difference of 0.0 PD (LoA: –4.0 and 3.9 PD).

In the ACT phase, the comparison between DCT and CCT (Fig 4D) showed a horizontal mean difference of –1.3 PD (LoA: –8.0 and 5.4 PD) and –1.9 PD in the vertical axis (LoA: –7.5 and 3.8 PD). Regression analysis similarly demonstrated a statistically significant proportional bias for both horizontal (slope = –0.28, *P* < 0.001) and vertical deviations (slope = –0.60, *P* < 0.001). The interobserver agreement in CCT for ACT (Fig 4E) showed a horizontal mean difference of –0.4 PD (LoA: –5.1 and 4.3 PD) and a vertical difference of –0.2 PD (LoA: –2.2 and 1.9 PD). Test–retest results for the DCT in the ACT phase (Fig 4F) produced a mean difference of –0.4 PD in the horizontal axis (LoA: –4.3 and 3.4 PD) and 0.0 PD in the vertical axis (LoA: –4.5 and 4.6 PD).

Repeated DCT measurements and interobserver CCT assessments demonstrated comparable LoA, although the LoA varied depending on the axis and test phase.

#### Intraclass Correlation Coefficients (ICCs)

Intraclass correlation coefficients were calculated to assess repeatability and agreement across all methods and conditions (Table 2). Intermethod ICC analyses confirmed strong agreement between DCT and CCT measurements.

**Table 2:** Intraclass Correlation Coefficients (ICCs) for Agreement and Repeatability of Ocular Deviation Measurements Obtained with the CCT and the DCT

Method	Test Phase	Deviation Type	ICC	95% CI
CCT vs. DCT	CUT	Horizontal	0.93	[0.88–0.96]
		Vertical	0.81	[0.68–0.89]
	ACT	Horizontal	0.96	[0.92–0.98]
		Vertical	0.81	[0.65–0.90]
CCT1 vs. CCT2	CUT	Horizontal	0.98	[0.97–0.99]
		Vertical	0.95	[0.93–0.97]
	ACT	Horizontal	0.97	[0.95–0.99]
		Vertical	0.96	[0.92–0.98]
DCT1 vs. DCT2	CUT	Horizontal	0.99	[0.98–0.99]
		Vertical	0.94	[0.89–0.96]
	ACT	Horizontal	0.99	[0.98–0.99]
		Vertical	0.93	[0.88–0.97]

ACT = alternate cover test; CCT = classic cover test; CI = confidence interval; CUT = cover–uncover test; DCT = DIVE Cover Test; ICC = intraclass correlation coefficient.

Intraclass correlation coefficients are reported for intermethod agreement between DCT and CCT, interobserver agreement in CCT (CCT1 vs. CCT2), test–retest repeatability of DCT (DCT1 vs. DCT2), separately for the CUT and the ACT, and for horizontal and vertical deviations.

Overall, these values indicate a high level of agreement between the digital and clinical methods, particularly for horizontal deviations, where agreement was excellent, and during the ACT condition. Interobserver agreement for the CCT was similarly high, while test–retest analyses demonstrated excellent consistency of the DCT across repeated measurements.

Taken together, these results demonstrate high repeatability of the DCT and strong agreement with the CCT across test phases and deviation axes.

### Correlation Analyses

**Figure 5:**
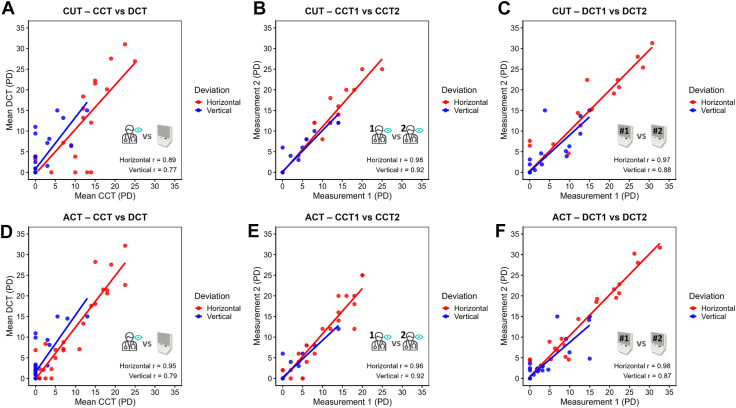
Pearson correlation analysis of ocular deviation measurements. Scatter plots show correlations between CCT and DCT measurements (**A**, **D**), between repeated CCT measurements by 2 observers (**B**, **E**), and between repeated DCT sessions (**C**, **F**). The top row corresponds to the CUT, and the bottom row to the ACT. Horizontal deviations are shown in red and vertical deviations in blue. Solid lines represent linear regression fits. Pearson correlation coefficients (*r*) are reported for each axis within each panel. ACT = alternate cover test; CCT = classic cover test; CUT = cover–uncover test; DCT = DIVE Cover Test; PD = prism diopters.

Pearson correlation analyses were performed to evaluate the linear association between measurements obtained with the DCT and the CCT, as well as between repeated measurements within each method (Fig 5).

In the CUT condition, strong correlations were observed between CCT and DCT measurements for horizontal deviations (*r* = 0.89) and moderate-to-high correlations for vertical deviations (*r* = 0.77). Interobserver correlations for the CCT were very strong (horizontal *r* = 0.98; vertical *r* = 0.92), while test–retest correlations for the DCT were similarly high (horizontal *r* = 0.97; vertical *r* = 0.88).

In the ACT phase, correlations between DCT and CCT measurements remained strong for horizontal deviations (*r* = 0.95) and moderate for vertical deviations (*r* = 0.79). Interobserver CCT correlations (horizontal *r* = 0.96; vertical *r* = 0.92) and DCT test–retest correlations (horizontal *r* = 0.98; vertical *r* = 0.87) were consistently high.

Across both test phases, correlations were higher for horizontal than for vertical deviations, and repeated measurements within each method showed stronger linear associations than intermethod comparisons, as expected given that intermethod comparisons incorporate additional sources of variability from both techniques.

## Discussion

Quantifying ocular deviation accurately remains one of the most technically demanding components of strabismus assessment. Although the prism cover test is considered the clinical reference standard, its execution depends heavily on examiner expertise, patient cooperation, and repeated manual occlusions. These factors are particularly challenging in young children and in patients with limited attention or reduced collaboration, where subtle deviations may be underestimated or inconsistently measured. Moreover, the absence of objective digital records limits reproducibility and longitudinal comparability.

In this context, the DCT was designed to address some of these intrinsic limitations. By integrating portable eye-tracking technology into a structured digital cover test protocol, the system enables objective and continuous quantification of eye deviation while presenting the task in a screen-based, game-like interface that may enhance engagement in pediatric populations. Unlike prism-based assessment, which relies on discrete measurement steps and subjective observation of refixation movements, eye tracking allows direct recording of gaze position and automated calculation of deviation magnitude, potentially reducing observer-dependent bias.

Against this clinical background, the main findings of the present study can be summarized as follows: (1) the DCT demonstrated high diagnostic accuracy for detecting ocular misalignment in both CUT and ACT phases; (2) agreement between DCT and CCT was good, particularly for horizontal deviations and during the ACT phase; and (3) repeatability of the DCT was excellent and comparable to interobserver agreement of the CCT. Together, these results support the feasibility of the DCT as an objective and reliable alternative to prism-based assessment.

### Diagnostic Performance Comparison with Previous Eye-Tracking Studies

A recent meta-analysis by Hartness et al^11^ reported pooled sensitivity and specificity values of 0.87 (95% CI, 0.79–0.92) and 0.83 (95% CI, 0.74–0.90), respectively, for automated technologies aimed at detecting ocular misalignment. In the present study, the DCT achieved comparable or higher diagnostic performance, with sensitivity and specificity of 85% and 98% in the CUT, and 92% and 87% in the ACT (Table 1). Sensitivity values were within the reported pooled 95% CI, while specificity in the CUT exceeded the upper bound of the corresponding 95% CI.

Direct comparison with individual studies further contextualizes these findings. Valente et al^15^ reported a sensitivity of 80% and specificity of 100%, whereas Chen et al^19^ described substantially lower sensitivity (47%) with moderate specificity (84%). In comparison, the DCT demonstrated a more balanced diagnostic profile, maintaining high sensitivity without compromising specificity across test phases.

Importantly, the present study provides a phase-specific analysis (CUT and ACT), which is rarely reported in previous automated approaches. Diagnostic performance and agreement metrics were consistently stronger in the ACT phase, reinforcing the relevance of sustained dissociation conditions for automated quantification of ocular deviation.

### Agreement and Correlation Analyses

Agreement between DCT and CCT measurements was good across test phases and axes, with small mean differences and clinically acceptable LoA (Fig 4). The Bland–Altman analysis provides the most informative assessment of agreement and allows visualization of trends across the range of measurements. Inspection of the plots suggested that differences between methods varied across the range of deviation magnitudes, although with considerable dispersion. Overall, CCT tended to yield slightly higher values at smaller angles, whereas DCT tended to yield higher values at larger angles. This trend was supported by regression analysis of the differences against the mean values, which demonstrated a statistically significant proportional bias in both test phases and axes. However, this pattern was not uniform across all subgroups and was less visually pronounced in some conditions, particularly in CUT horizontal measurements, where variability was greater. In horizontal ACT measurements, for example, LoA were ±6.7 PD, compared with values around ±11–14 PD reported in prior eye-tracking studies such as those by Yehezkel et al^16^ and Zou et al.^17^ These findings indicate that the agreement observed in the present study exceeds that reported in several previously published automated approaches.

Interobserver agreement for the CCT was strong, with narrow LoA across both phases and axes. Previous reports have described interobserver variability of up to approximately 12 PD as acceptable in prism-based assessment, particularly for moderate-to-large deviations.^9^^,^^31^ The consistent performance of the clinical reference standard in the present cohort supports the validity of the intermethod comparison and reinforces that the observed agreement reflects the reliability of the DCT.

Repeatability of the DCT was similarly stable. The standardized digital protocol—fixed testing distance, predefined occlusion sequence, uniform stimulus presentation, and automated gaze quantification—likely contributed to the low variability observed across repeated measurements. Test–retest analyses showed mean differences close to zero and LoA of approximately ±4 PD, indicating within-method consistency comparable to that of experienced clinicians while benefiting from procedural standardization.

These results were reinforced by ICC analyses (Table 2). Intermethod ICCs ranged from 0.93 to 0.96 for horizontal deviations and were slightly lower for vertical deviations, remaining within the range reported in the meta-analysis by Hartness et al^11^ (pooled ICC 0.92; 95% CI, 0.77–0.98). Test–retest ICCs for the DCT were consistently ≥0.93, confirming excellent relative reliability. Taken together, these metrics indicate that the DCT achieves agreement with the clinical standard comparable to that reported for other automated systems, while maintaining strong internal repeatability.

Pearson correlations were consistent with these findings (Fig 5), showing strong linear associations between methods—particularly for horizontal deviations—and very high within-method correlations. As expected, correlation coefficients were higher for horizontal than for vertical components. Although correlation alone does not establish agreement, these findings are consistent with the agreement metrics described above.

### Horizontal versus Vertical Deviations

Agreement between methods was consistently stronger for horizontal than for vertical deviations. This pattern was not due to poor vertical repeatability of the DCT, as test–retest analyses demonstrated stable vertical measurements within the digital method. Rather, the lower intermethod agreement in the vertical axis likely reflects inherent challenges in clinical assessment of vertical components.

Few previous eye-tracking studies have reported agreement separately by axis, and most include limited numbers of patients with vertical deviations. In the present cohort, vertical deviations measured with the DCT tended to be slightly higher than those obtained with the CCT, suggesting a systematic difference rather than random variability.

Clinically, vertical deviations frequently coexist with horizontal components (100% in our cohort), requiring simultaneous prism compensation in both axes. This may lead to underestimation of the vertical component during routine prism-based assessment. In contrast, the DCT derives vertical deviation directly from continuous gaze data and is not constrained by discrete prism steps. These methodological differences provide a plausible explanation for the observed vertical discrepancies and support the interpretation that the digital measurements are stable, while potentially capturing components that may be underestimated with conventional testing.

### CUT versus ACT Performance

Agreement between methods was generally stronger during the ACT than during the CUT. This pattern likely reflects intrinsic differences between phases rather than reduced validity of the digital CUT.

In the CUT condition, small deviations were sometimes slightly higher with the CCT, whereas larger deviations tended to be higher with the DCT. For small angles, repeated manual occlusions in clinical practice may increase dissociation and reveal slightly larger deviations. For larger angles, the continuous scale of the DCT may allow more precise quantification than discrete prism steps, which can result in rounding to the nearest lower increment.

During the ACT, sustained alternation prevents fusion in both methods, creating more comparable dissociation conditions and reducing variability between techniques. In addition, as ACT was performed after CUT, increased fatigue may have facilitated fuller expression of latent components, further aligning measurements.

Importantly, assessing both phases remains clinically relevant. The CUT reflects manifest deviation under brief dissociation, whereas the ACT captures latent components and full misalignment under sustained dissociation. The DCT allows objective quantification of both, preserving this clinically meaningful distinction.

### Clinical Implications

The DCT may be particularly useful in settings where examiner experience or patient cooperation is limited, such as pediatric clinics or high-demand clinical environments. Its standardized protocol and digital output facilitate longitudinal monitoring and may reduce interexaminer variability in routine practice. These features support its potential role not only as a complementary tool to prism-based assessment, but also as a step toward more standardized ocular alignment evaluation.

### Limitations and Future Directions

While this study provides validation of the DCT across test phases and axes, some limitations should be acknowledged. The present study focused on measurements in primary gaze to ensure a standardized and time-efficient assessment. Evaluation across multiple gaze positions and detailed characterization of incomitant strabismus were beyond the scope of this protocol. The number of participants with vertical or mixed deviations was limited, which may affect the precision of subgroup analyses. In addition, participants with deviations greater than 25 PD were not included, which may limit generalizability across the full spectrum of ocular misalignment. Larger cohorts, ideally within a multicenter design and including a broader range of deviation magnitudes, would allow more granular performance assessment across deviation subtypes (e.g., esotropia, exotropia, and mixed deviations) and magnitude ranges (e.g., <25 PD vs. > 25 PD). Sex-based analyses were not performed because the study was not powered to detect sex-related differences.

All measurements were performed at near distance (65 cm), which was selected to optimize eye-tracking accuracy. Although the system could potentially be adapted for other near distances (e.g., 45–75 cm), performance at longer distances was not evaluated in the present study. Assessment at true distance fixation was beyond the scope of this validation and would require further technical adaptation.

Manual occlusion was still required, and although standardized, may introduce minor variability. Further multicenter studies, broader pediatric populations, and evaluations in real-world clinical environments will help clarify the generalizability and scalability of this approach.

In conclusion, the DCT demonstrates high diagnostic accuracy, strong agreement with the clinical reference standard, and excellent repeatability across test phases and deviation axes. Its performance exceeds most reported in previous eye-tracking–based approaches, while offering additional advantages in terms of portability, objectivity, and phase-specific characterization of ocular deviations. Together, these findings support the DCT as a robust and clinically meaningful tool that may contribute to a more standardized, objective, and scalable assessment of ocular alignment in routine practice.
